# Parents' perspectives on user participation and shared decision‐making in adolescents' inpatient mental healthcare

**DOI:** 10.1111/hex.13443

**Published:** 2022-02-07

**Authors:** Stig Bjønness, Trond Grønnestad, Jan O. Johannessen, Marianne Storm

**Affiliations:** ^1^ Department of Public Health, Centre for Resilience in Healthcare (SHARE), Faculty of Health Science University of Stavanger Stavanger Norway; ^2^ Department of Public Health, Faculty of Health Science University of Stavanger Stavanger Norway; ^3^ Department of Psychiatry Stavanger University Hospital Stavanger Norway

**Keywords:** adolescent, inpatient, mental healthcare, parents, qualitative study, shared decision‐making, user participation

## Abstract

**Background:**

Parents are a resource that can be of considerable importance in supporting their adolescents' recovery and shared decision‐making processes. However, involving both adolescents and their parents in treatment creates challenges. Understanding the roles of all decision stakeholders is vital to the implementation of shared decision‐making and delivery of high‐quality healthcare services.

**Objective:**

The aim of this study is to explore parents' experiences with adolescents' participation in mental health treatment and how parents perceive being involved in decision‐making processes.

**Design:**

This was a qualitative study with a phenomenological, inductive design. Content analysis of data from qualitative interviews was performed.

**Setting and Participants:**

This study took place in a Norwegian public healthcare setting. Twelve parents of adolescents who received treatment for severe mental illness participated.

**Results:**

Four themes were identified: (1) self‐determined treatment, but within limits; (2) the essential roles of parents; (3) the need for information and support; and (4) the fight for individualized treatment and service coordination.

**Conclusion:**

User participation is vital in adolescent mental healthcare and parents play essential roles regarding the shared decision‐making process. However, user participation and shared decision‐making pose several dilemmas. Parental involvement in treatment decisions may be necessary when adolescents are mentally ill, but could simultaneously hinder those adolescents' empowerment and recovery. Cooperation among parents, adolescents and healthcare professionals can improve treatment engagement and adherence, but may be challenged by divergent interests. Health services should provide family‐oriented services to utilize the potential of parents as a resource and minimize conflicting interests.

**Patient or Public Contribution:**

Two adolescent user representatives participated in designing the study.

## 1 BACKGROUND

Although parents could play a significant role in supporting adolescents through mental health crisis and recovery, the hospitalization of adolescents is also a time of crisis for their parents. Parents' involvement and contact with healthcare professionals influence their ability to care for their children. To help parents provide the necessary support, they should be afforded informational, emotional and instrumental support.^1^, ^2^ The relationship and collaboration between parents and healthcare professionals in adolescents' inpatient units are crucial for adolescents' recovery from mental health disorders.^3^ However, adolescence is a distinctive developmental period requiring tailored clinical approaches.^4^ Therapists face the challenging task of involving adolescents and parents with competing and divergent perspectives about treatment, thus risking the therapeutic alliance with the adolescents if they do not agree with their perspective.^5^ The adolescents' sense of autonomy and relationship with the therapist during hospital admission is crucial for participation in treatment.^6^

User participation addresses patients' involvement in their treatment, including their influence in decision‐making.^7^ Shared decision‐making is ‘a process in which clinicians and patients work together to select tests, treatments, the management or support packages, based on clinical evidence and the patient's informed preferences’.^8^ Shared decision‐making improves patients' involvement, knowledge, coping skills, satisfaction and treatment adherence^8^, ^9^ and has long been cited as an ideal model for treatment decisions.^8^, ^10^ User participation and shared decision‐making are connected to empowerment.^11^ Empowerment consists of the conditions that make patients ‘willing and able’ to assume an active role in their care and to participate in decisions.^11^, ^12^ The process by which individuals gain control over their own lives is described as empowerment; greater user participation increases empowerment.^12^

Family members and parents are often involved in decision‐making about treatment,^10^ and previous research indicates a decision‐making power shift from professionals to families.^13^ According to research, parents can play a significant role in shared decision‐making processes and supporting adolescents' recovery.^1^, ^2^, ^14^ Parents' roles can include advising, negotiating on their children's behalf and supporting and reinforcing the treatment decision as caretakers.^10^

Acknowledgement of the family perspective and information sharing are factors reported to influence shared decision‐making in child and adolescent mental health services (CAMHSs).^15^, ^16^ A scoping review^17^ suggests that the usage and implementation of decision support interventions and parents' involvement are influenced by time, accessibility and the appropriateness of the interventions. Previous research^18^ found more patient‐reported improvement in mental health symptoms associated with shared decision‐making. However, improvement in mental health difficulties was only found when both parents and patients reported a high level of experience of shared decision‐making. These studies did not focus on inpatient mental health treatment. Yet, despite the importance of combining the perspectives of patients, parents and professionals, researchers have paid little attention to user participation in inpatient CAMHS treatment and parents' perspectives on shared decision‐making.^13^, ^17^ Clarity about what shared decision‐making constitutes is essential for implementing and assessing shared decision‐making in adolescent mental health.^19^ Further research is needed to support parents as stakeholders in shared decision‐making to create engagement opportunities for patients and families and enable health services to benefit from the experiences.^14^

This paper generates knowledge about user participation and shared decision‐making in CAMHS from parents' perspectives. Our objectives were to explore parents' perspectives on adolescents' participation in inpatient mental health treatment and to explore parents' role in the shared decision‐making processes.

## 2 METHODS

This qualitative study had a phenomenological, inductive design. It explored the experiential aspects of a phenomenon. Parents' perspectives were examined and analysed without being shaped by theory. Nevertheless, qualitative research is influenced by a theoretical framework.^20^, ^21^ Alongside the phenomenological approach, this study considers empowerment as a theoretical framework for shared decision‐making.

### 2.1 Study setting and participants

The study setting was public mental health services for adolescents in Norway. CAMHSs provide healthcare for patients up to 18 years of age. Adolescents are defined as individuals older than 13 years of age. Acute inpatient clinics provide services for adolescents with severe mental disorders. Acute admissions are intended to be stabilizing, preferably voluntary and short. However, admissions varying in length and coercion are occasionally used. Outpatient treatment is the most utilized mental health service and is usually provided before and/or after admission.^22^ In addition, some treatment and rehabilitation units offer ward and recovery‐oriented services to young adults from the age of 16. Multifamily group counselling is a common service offered, where several families with similar health problems meet regularly with two therapists. The age of sixteen years is considered the legal age for medical decisions in Norway; for patients younger than 16 years of age, their parents must consent to treatment.

Inclusion criteria were parents of adolescents with a severe mental illness (e.g., psychosis and suicidality) who had received inpatient mental healthcare before the age of 18. Study participants were parents of adolescents with an average age of 17.5 years. Most of the adolescents had several admissions to inpatient treatment. Hospitalisations ranged from 1 week to more than a year, averaging four months. Some adolescents were admitted at the time of the interview; others were discharged and received outpatient treatment. Information about the participants and recruitment is provided in Table 1.

**Table 1:** Description of participants and recruitment

Participant ID number	Parenting role	Recruited from	Adolescents' gender/age	Form of interview	Received family counselling
1	Father	Inpatient unit	Girl/16	Individual	No
2	Mother	Inpatient unit	Boy/22	Individual	Yes
3	Mother	Inpatient unit	Boy/22	Individual, digital	No
4	Mother	Inpatient unit	Girl/18	Couples	No
5	Father	Inpatient unit	Girl/18		No
6	Mother	Outpatient unit	Girl/21	Couples, digital	Yes
7	Father	Outpatient unit	Girl/21		Yes
8	Mother	Outpatient unit	Girl/18	Individual, digital	No
9	Mother	Family group	Girl/15	Individual	Yes
10	Mother	Outpatient unit	Boy/13	Individual	No
11	Mother	Outpatient unit	Girl/15	Individual, digital	No
12	Mother	Family group	Girl/15	Individual	Yes

### 2.2 Recruitment

To arrive at an in‐depth understanding of parents' perspectives, we used purposive sampling to include study participants with experience to inform an understanding of the central phenomenon.^23^ We informed ward leaders from two CAMHS acute inpatient units (13–18 years) and two treatment clinics with inpatient and outpatient treatment for young adults (16–24 years) about the study. All recruitment sites were part of hospital mental healthcare services providing care for severe mental disorders. The therapist responsible for treatment received written information about the study and method and where to recruit parents who fulfilled the inclusion criteria to participate in an interview. Eligible parents were handed out written information about the project by the therapist. Those who were interested in participating agreed that the therapist could share their contact details with the researcher (S. B.), who then contacted them via email or phone to schedule time for an interview.

### 2.3 Data collection

The interview guide was informed by published literature^13^, ^19^, ^24^ with open‐ended questions, based on Kallio, Pietilä, Johnson and Kangasniemi.^25^ Table 2 presents the main questions in the interview guide. To increase understanding, a workshop with two adolescents with user experience supplemented the interview guide by reviewing and adding questions. We conducted one pilot interview and made some minor adjustments to the follow‐up questions. Twelve parents were interviewed between August 2019 and October 2020. Some parents preferred to be interviewed with their partners, so two interviews were conducted with couples. The other interviews were conducted individually. The interviews were scheduled to last 1 h, and ranged from 37 to 90 min; they averaged 61 min. The participants could choose where and how the interviews were to be conducted. Four interviews were carried out over Zoom or Skype, two were conducted at participants' homes and the remaining four interviews were held at the treatment units. One interview was conducted by author T. G.; the rest were conducted by the first author S. B. All interviews were audio‐recorded and transcribed into text by S. B. The interviews were conducted in Norwegian, and quotes were translated into English by S. B. and proofread by a professional editor.

**Table 2:** Interview guide

**Introduction/background**	
1	Can you first briefly describe the family?
2	Can you tell me a little about the contact with mental healthcare?
3	What kind of expectations did you have?
4	To what extent are you satisfied with the information you have received?
**Collaboration, participation and shared decision‐making** (describing SDM for participants)	
5	How do you experience being involved in the treatment and decisions regarding the treatment?
6	How does your son/daughter feel about the therapist involving you?
7	How would you describe the relationship/collaboration between:
	‐ your son/daughter and his/her therapist/primary contact?
	‐ inpatient clinic, outpatient clinic or other wards
	‐ others (school, child welfare, GP, municipal healthcare services)
8	What do you think about user participation for your son/daughter and whether he/she should be involved in making treatment decisions?
9	Do you have any thoughts on what it takes to be involved in deciding on their own treatment?
10	What do you think your role as a mother/father should be when decisions are made related to the treatment?
**Participation and decisions related to diagnoses, treatment and planning**	
11	How have you experienced the process of assessing mental illness/diagnosis?
12	Do you know if your son/daughter has received one or several diagnoses?
13	If your son/daughter has received medication, how was his/her participation in that decision and your thoughts about it?
14	Are you familiar with the treatment plan, and do you have the opportunity to influence it?
15	Do you have any experience with coercion/involuntary treatment? If so, what are your thoughts about it?
**Winding‐up**	
16	In which areas do you think it is important that young people participate/have a say in decision‐making?
17	What do you think, as a mother/father, is the most important thing about the healthcare service?
18	Do you have any questions, or is there something else you think is important to add?

### 2.4 Data analysis

A qualitative thematic content analysis was used to analyse the interview data.^20^ We used an inductive ‘bottom‐up’ analysis process to establish codes  and themes to capture the essence of the interview participants' views.^20^, ^26^ The analysis was conducted in six steps, as described by Braun et al.^20^

Step 1. Transcribed interviews were read several times for the authors to familiarize themselves with the data and attain a general understanding.

Step 2. All authors created codes on meaning units in the text. The codes were designed as phrases evoking relevant features of the data.

Step 3. Collaborative discussions were held of first impressions and collation of similar codes into coherent clusters with preliminary themes.

Step 4. The preliminary themes were reviewed in relation to the coded and organized meaning units. A clearer understanding of the themes emerged as we checked the themes across the whole data set.

Step 5. The authors collaboratively adjusted and named the themes and ensured that they were coherent, addressed the research question and reflected the lived experience in the original transcripts.

Step 6. The results were reported in a final analysis with extract examples. Table 3 presents data extracts, codes and themes from the analysis.

**Table 3:** Example of analysis

Data extract/meaning unit	Coded for	Theme
I think it is important that they are heard. If they are pushed, they will resist and clearly not benefit so much from treatment. But I think it's a bit scary that they should have the last word in all decisions with their sick mindset. It is about that balance…	Should be heard	Self‐determined treatment, but within limits
	Reduced ability to decide	
One reaches a point where one can no longer cope, but the young ones still need our support and confirmation. I think maybe there should be services that include us as well. Although it requires more resources, I think it would prevent many problems in the long run	Parents also need help	To be supportive, we need information and support
	Services for relatives prevent problems	

### 2.5 Research ethics

The study was approved by the Norwegian Regional Ethics Committee (2017/1195) and the health trust's privacy representative (ID669). Voluntary participation was emphasized, and all participants provided informed written consent. The researcher provided written and oral information about the study and the opportunity to withdraw their consent without consequences. The interviewers had no connection to the participants, and the participants chose whether the interviews should take place at the clinic or elsewhere. All data were treated confidentially, and no information was exchanged between the researchers and the clinics. Whether the parents chose to participate or withdraw their consent had no consequences for them or the health services that the adolescents received.

## 3 RESULTS

The analysis resulted in four themes of the parents' experiences with adolescents' participation and their role in the shared decision‐making processes: (1) self‐determined treatment, but within limits; (2) the essential roles of parents; (3) the need for information and support; and (4) the fight for individualized treatment and service coordination.

### 3.1 Self‐determined treatment, but within limits

Self‐determined treatment was likened to having both hands on the steering wheel and taking ownership of one's healthcare and treatment. This theme reflects the dilemmas that the parents expressed. The parents referred to self‐determined treatment as adolescents' ability to make decisions and take responsibility. By achieving this, the adolescents would engage themselves in their care, become independent and able to take care of themselves as they matured. Such independence was emphasized by parents as the ‘right thing’ and as the ideal they wanted for their children. According to the parents, health services strived for voluntary treatment and adolescents' participation. The approach harmonized with their values and, according to the parents, yielded the best treatment effect.

*I experience that she agrees and participates in decisions herself (…) Crucial for success is that they agree and are ready to contribute to the treatment*. (Parent 1)

However, letting adolescents make their own treatment decisions posed several challenges. Some adolescents were unable to express a need for help, did not want help, lacked insight into their mental health or covered up their difficulties in contacting healthcare professionals. The parental dilemma was the extent to which they should strengthen their adolescents' autonomy by allowing them to make decisions. At the same time, this risked prolonging the mental illness and its consequences. The parents handled this in a variety of ways. Some took the initiative to arrange treatment without informing the adolescent. As one mother said, despite her desire to engage her son and leave the decision to him, she had no choice but to go behind his back to initiate inpatient treatment. Several parents explained that their sons or daughters were relieved by not having to take the initiative to hospital admittance.

*It's like a double‐edged sword. I have experienced that she often says no, but she is actually happy about it. It just takes a while before she sees it for herself*. (Parent 9)

Several parents claimed that treatment onset and admission decisions should not be left to adolescents with psychosis, very depressed thought patterns or severe mental illness. According to these parents, the concept of consent was too broad. Hence, in some situations, the parents saw coercion as unavoidable. The parents depended on the healthcare professionals to assess the adolescents' maturity, insight and the severity of the mental illness. However, further in the treatment, the parents described it as crucial that they left decisions to the adolescents without interfering. They tried to balance their involvement so that their son or daughter would gradually take more responsibility.

Although the parents claimed to know what was best for their children, they admitted that the treatment had poor utility without the adolescents having a say in decision‐making. If the parents made the decisions, there was a risk that their adolescents would oppose and fail to comply with the treatment, and resist self‐determination later on. Furthermore, as the adolescents became older, parents' involvement depended on the adolescents' consent. Several parents sought a balance between proper healthcare and allowing adolescents to be in control of decisions. One mother described the process of gradually handing over the responsibility to her son:

*We have learned to keep our hands behind our back… The most challenging thing during admission was to let him participate in the treatment without influencing him about what is best. We must leave the choices to him. It's his role, not mine, although I fear and dread of where it might lead him. It's terribly difficult not to be part of decisions, even though you know you must leave it to him*. (Parent 2)

### 3.2 The essential roles of parents

This theme describes parents' role before the decisions and in supporting the adolescents through treatment and recovery. Although the parents did not believe that the final decisions in shared decision‐making should be left to them, they stated that they played an essential role in the shared decision‐making process. Parents often found themselves in situations where they held multiple roles, such as therapist, teacher, friend and coordinator. They recognized that this was not consistent with the parental role that they should or wished to possess. Moreover, many adolescents did not want to share everything with their parents. Nevertheless, the parents had experienced holding an important role in facilitating treatment and shared decision‐making. They found solutions and negotiated on behalf of the adolescents so that they received suitable treatment. Adolescents' participation in shared decision‐making required treatment alternatives, and the parents did their utmost to ensure access to services that could contribute to recovery. Several parents also looked for suitable therapists and helped convey to them what they considered appropriate treatment and measures for their sons and daughters.

*The parents are the ones who know their child best. It is a new health professional who enters. They don't know how this person reacts and what is important for her. So dialogue with parents is essential! And since she doesn't talk much, we have had to tell them what is relevant for them to know*. (Parent 6)

Carrying the adolescents' voices and opinions was considered necessary throughout the treatment as they often approached their parents about topics they were unable to advocate for themselves. Typical examples were topics that the adolescents found embarrassing or areas of disagreements with the therapist. After treatment decisions had been made and treatment initiated, the parents emphasized their significance as they observed its effects and functional decline or improvement. Some parents pointed out that they became responsible for their adolescents' medication adherence.

*The parents are the ones who see changes in their kids because they know how they usually are over a more extended period of time. Therefore, parents should be involved and asked how the treatment is working*. (Parent 7)

Parents contributed by helping their adolescents become confident in their role as patients, building confidence in the therapist and in treatment to increase participation. Most importantly, the parents emphasized their supportive role. Without their encouragement, the adolescents would easily give up or refuse to commit to the treatment.

*The significance of parental support is underestimated. Parents are a resource in a slightly different way. Our resource is to be able to cheer them on, so to speak. ‘This was good, so much you have learned, this has done you good’… We have to trust the healthcare services rather than questioning their reliability. I think scepticism closes the doors*. (Parent 2)

This statement points to another key element. To build and pass on trust, the parents themselves needed confidence in the healthcare services. Most parents had trust in the therapists' expertize at the onset of the treatment. What affects trust and how this developed are described in Theme 3.

### 3.3 The need for information and support

Some parents said that they initially thought that the healthcare professionals knew best, but gradually their confidence declined. Other parents claimed that their confidence in healthcare professionals increased with time. Trust in the health services developed according to the parents when receiving information and experiencing being involved. Several parents reported that they had not received information because their sons or daughters did not want them involved in the therapy. They wondered why they could not receive general information about mental disorders, treatment and how to interact with their sons and daughters during treatment. This lack of information limited their ability to contribute to and support the adolescents in decision‐making. In the words of one parent:

*It is difficult to advise, recommend something, or push in a direction when one does not know anything about the alternatives. It is challenging to have a dialogue to motivate and accept something when you do not know what the choices consist of*. (Parent 3)

One mother explained the difference that she had found between somatic hospitalization, where the parents were routinely involved, and mental healthcare, where confidentiality issues precluded their involvement. Several parents requested routines for parental information. The parents also stated a need for support and advice or counselling. To avoid intervening with the therapy, it was suggested that guidance to parents could be provided by a healthcare professional other than the adolescent's therapist.

*Parental guidance is essential. We realized that here we needed help to be parents. One enters such a mode where we do not have the opportunity to process everything because one does not know what is coming around the next corner*. (Parent 11)

Being the parents of a severely mentally ill adolescent was described as a crisis for the whole family. Several parents told stories about being exhausted and becoming disabled or ill themselves. Although the adolescents' hospital admission could bring some relief, the responsibility to care for the adolescents was eventually handed back to the parents. Some parents struggled with not being informed and involved as a collaborative partner when the adolescent turned 18 years old. They found this challenging as they still had the care and financial responsibilities. Parents' feeling of being left abandoned made it difficult for them to support their adolescents. The parents emphasized a need for family interventions. Those who had participated in multifamily groups found it of great significance.

*It has been a long process to understand what the disease entails. I feel that being part of a family group helped to get some of those answers. Together with other families in the same or similar situations, we could share experiences*. (Parent 7)

### 3.4 The fight for individualized treatment and service coordination

According to the parents, user participation presupposed time for the healthcare professionals to understand the problem, not jumping to conclusions or strictly following guidelines. To do so entailed listening to the adolescents' needs, informing them about medications, their effects, benefits and side effects and giving them the time to consider the pros and cons of medications. The parents wanted their adolescents to receive individually tailored treatment, and several had had to fight for their adolescents.

*We often experience that they try to use one success story and put it on a second child without asking. Observe, explore what's right for them. It is time‐consuming and more expensive, but you get poor results if you do hasty work*. (Parent 5)

Some parents described acute clinics as solely designed to limit harm and missed alternative and continuous services for severe ill adolescents under 18. As one mother said, her daughter could not participate in decisions when she had not spoken before discharge was scheduled. The parents had several suggestions to increase user participation. They suggested that shared decision‐making for adolescents should be concerned not only with defined treatment options but also with how the treatment should be administered. They thought that it was just as important for adolescents to choose how the treatment should be carried out. Suggestions included therapy sessions outside the clinic, at school or home. Several parents argued for a combination of therapy with activities where the adolescents did not have to sit in front of the therapist. Parents viewed decision‐making as difficult for some adolescents.

*One of the tough things for her, and it has to do with her illness, is to make decisions. So, to give her utterly open outcome spaces makes it entirely impossible for her. It is impossible in trivial things, so to give her all the options… She would not be able to answer. But if she could get a kind of delimitation, choose between this and this, then she would probably more easily be able to participate in decisions*. (Parent 11)

The adolescents had complex difficulties that required compound services. Several parents pointed out that they had to coordinate their son's or daughter's health services themselves. Parents found it stressful to handle communication and information exchange between professionals across the hospital, outpatient clinic, GP, school and work practice. When decisions were made and treatment was implemented in the inpatient clinic, it could be a struggle to continue them after discharge. The parents gave examples of their children being discharged without notice from acute clinics and without time to prepare for the transition to municipal services. Thus, decisions and treatment initiated in the inpatient clinic were not continued after discharge. Parents insisted that a professional coordinator was necessary.

*We had to be the mediator between them (different healthcare services) on things they ought to know. It is silly because it takes a lot of energy, and it has taken a long time for us to understand the system. Who is really responsible, and who should take the initiative? In the end, we have to do it*. (Parent 9)

The parents were not particularly concerned with diagnoses. Several of them thought of diagnoses merely as guidance, and some said it could contribute to proper treatment. Some parents found diagnoses helpful in the sense that they provided an understanding of what the young people were contending with. At the same time, most emphasized that diagnoses must not be at the expense of individually tailored treatment. From the parents' perspective, the most important thing about diagnoses was that they affected the kind of help they were entitled to.

## 4 DISCUSSION

This study examines user participation and shared decision‐making in treatment for adolescents with severe mental illness from their parents' perspective. The study offers some insight into the challenges and dilemmas that parents face with their adolescent's participation in treatment. Although adolescents and the therapist are the main actors in shared decision‐making, parents also have an essential role in the decision process. They provide and share necessary information, support the adolescents, contribute as mediators and influence treatment follow‐up. To make the best use of parents as resources, parent‐ and family‐oriented services are required. In the extension of inpatient treatment, individualized and compounded healthcare requires the coordination of services.

Shared decision‐making is often considered as a process between patients and therapists that relies on decision tools.^8^, ^27^ A systematic review^19^ providing an overview of shared decision‐making models found that most studies were from somatic and stated that the process may differ by healthcare setting. Our study did not explore the use of decision tools. Nevertheless, the results are in line with research among adolescents with physical health problems where the decision‐making process is described as situational and adolescents' participation depends on parents and health professionals.^28^ Previous research has identified the collaboration between the treatment clinics and parents as a predictor for quality of care. The literature emphasizes consideration of parents' needs and providing them with enough time to support and inform.^29^ Our study highlights healthcare professionals' collaboration with parents as they provide the kind of information and communication between adolescents and therapists that promotes user participation and shared decision‐making. Parents' role in adolescents' decision‐making has been reported in previous studies, suggesting that parents' support helps their adolescents to benefit from shared decision‐making.^16^, ^17^, ^18^ According to our results, parents, therefore, play a key role in the information exchange that underpins shared decision‐making.

A treatment approach that embraces shared decision‐making promotes adolescents' participation and engagement in treatment and should continue after inpatient care.^1^ Most adolescents depend on their parents after discharge from inpatient care. Our findings suggest that if parents support user participation and shared decision‐making, their adolescents are more likely to stick to the interventions. A review^27^ identified favourable treatment outcomes when parents were involved. Fewer decisional conflicts and increased maintenance of recommended treatment were found. However, the same review reported research with no difference in outcome with parents engaged in approaches to facilitate shared decision‐making. Lack of support from healthcare professionals was suggested as a possible explanation.^27^ Our study supports this explanation. Parents initially trusted their children's healthcare professionals. Those parents who used family‐oriented services with information and support maintained their trust, while those who felt excluded expressed scepticism about the treatment. The significance of family support and its relation to treatment and recovery have been established.^1^, ^18^, ^29^ The more the parents learn about their adolescents' symptoms, the more support and understanding the adolescents receive.^30^ The parents emphasized that participation in multifamily groups significantly supported the family and the adolescent's recovery. Severe mental illness among adolescents is a burden for the whole family, and the parents need support through the crisis to maintain care.^31^ As parents gain more insight, their sense of empowerment and capacity to manage the situation increases.^13^

The participants in the study expressed concern about the ability of mentally ill adolescents to make informed decisions. Whether adolescents are capable of being involved in decision‐making has been given much attention in research. One study has found that adolescents have the capacity, desire and will to participate in shared decision‐making.^27^ However, little research has examined both adolescents with severe mental illness and their parents' perspectives.^17^ Our results highlight the parental dilemma in terms of the extent to which parents should leave decisions to the adolescents to strengthen their autonomy and empower them, although the parents also claimed that mentally ill adolescents with a lack of insight could be incapable of making responsible decisions. The parents expressed concerns about risking prolonging symptoms and the mental illness, especially regarding treatment onset. Impaired function over time in adolescence has long‐lasting consequences for health, education and social networks.^32^ Nevertheless, the parents in our study acknowledged the importance of gradually relinquishing control and choices to adolescents. Empowerment enables people to take charge of their own lives and make decisions,^12^ in line with the parents' wishes for their sons and daughters in our findings. This is also relevant to the treatment outcome as adolescents consider empowerment an essential factor in their recovery.^33^

Previous studies have found a need for individualized and customized treatment and a link between user participation and adolescents' sense of empowerment.^6^, ^15^ Adolescents with serious mental illness face challenges in the continuity of care and transition as they move between care settings.^34^ The parents in our study noted a lack of information exchange and integration of the tailored treatment when dealing with other health and welfare services. The results revealed a need for support from healthcare professionals that provides guidance through the mental health, school and welfare systems. Parents depend on counselling to meet the needs of their ill adolescents and maintain their health.^31^ The call for effective coordination and support for caregivers has been addressed in previous research and is associated with better service utilisation, social functioning and quality of life.^34^

Engaging adolescents in their treatment and empowering them is desirable. However, the degree of autonomy in decision‐making will vary according to the severity of the disease and the risk of injury.^35^, ^36^ Simmons and Gooding^36^ describe supported decision‐making as a broader approach that encompasses shared decision‐making, emphasizes the patient as the final decision‐maker and forms an alternative to substituted decision‐making and paternalism. In this context, adolescents are not considered ‘purely’ autonomous, but can make decisions with parental support without parents taking over the decisions.^35^, ^36^ The balance between parents' overinvolvement or exclusion from involvement in an adolescent's treatment is essential. Both extremes hinder user participation and adolescents' empowerment and can be linked to expressed emotions. Expressed emotions refer to particular emotions, attitudes and behaviour expressed by relatives to mentally ill patients.^37^ Strongly expressed emotions such as overinvolvement and criticism can reduce collaboration and symptom improvement.^31^ Hence, it is vital to understand how to balance parents' involvement.

However, the involvement of parents in adolescents' treatment is not straightforward. The parents may have played a role in the origin of the adolescents' mental problems, and adolescents emphasize that they should control the parents' involvement.^6^ Healthcare professionals acknowledge that they sometimes must accept that the adolescents refuse to involve their parents to establish a treatment alliance.^5^ Parents and adolescents may have opposing expectations and interests, and privacy is a barrier to collaboration between parents and professionals.^13^ Our study findings suggest routines to give parents general support and information without compromising privacy or the therapist–patient alliance. Based on the results, we argue that user participation can be considered as a spectrum. The spectrum ranges from a low to a high degree of what our study participants referred to as self‐determined treatment. Substituted decision‐making is considered a low degree of user participation and shared decision‐making that emphasizes the adolescent as a final decision‐maker is considered a high degree of user participation.

### 4.1 Limitations and strengths

Initially, the recruitment strategy was to invite participants through CAMHS inpatient units. The recruitment strategy was changed during the study due to COVID‐19, as we faced difficulties with accessing the units and recruiting. Therefore, we approached several clinics and treatment services with a request for recruitment. The participants are recruited from different treatment clinics and represent both adolescent and young adult patients. Some of the patients were admitted to acute clinics, others to rehabilitation clinics and some were only receiving outpatient care. However, all the parents were still selected purposefully ensuring experience from inpatient CAMHS which they were asked to reflect on in the interviews. Still, it is considered a limitation that four of the participants were parents of adolescents with current age over 18 years and representing experiences with both CAMHS and mental healthcare for young adults. Procedures for audio‐recording and transcription were performed similarly in digital interviews, and image transfer minimized potential inequality between in‐person and digital interviews. Even though digital interviews were offered to prevent the risk of COVID‐19 infection, it can be considered a strength that participants themselves could choose their preferred form of interviews. Parts of the analysis were conducted separately by the research team and then discussed in joint analysis meetings to limit the influence of researchers' preconceptions. It can be considered as a limitation that the study did not include the perspectives of the adolescents themselves. We have conducted a separate study on adolescents' perspectives.^6^

## 5 CONCLUSIONS AND IMPLICATIONS

We have explored parents' perspectives on user participation and shared decision‐making for adolescents in mental healthcare. The study provides insight into parents' dilemma with user participation. The findings and their implications will enable healthcare organisations to learn from their experiences. User participation contributes to empowerment, and shared decision‐making reflects a high degree of user participation. Parents hold several essential roles that affect shared decision‐making for adolescents. They are liaisons between the therapist and the adolescents, can help the adolescents reflect on alternatives and, by supporting them, increase treatment adherence. Parents need information, guidance and support to maintain these roles. Parental insight and professional coordination of services facilitate adolescents' engagement and empowerment. Health services should provide family‐oriented services to maximize the potential of parents and minimize conflicting interests. In situations where parents cannot be directly involved in the therapy for reasons of privacy or conflicts, routine services that provide parents with general information and guidance on mental disorders and treatment are recommended.

## CONFLICT OF INTERESTS

The authors declare that there are no conflicts of interest.

## Data Availability

The data that support the findings of this study are available on reasonable request from the corresponding author. The data are not publicly available due to privacy or ethical restrictions.
